# The Association Between Risk Perception and COVID-19 Vaccine Hesitancy for Children Among Reproductive Women in China: An Online Survey

**DOI:** 10.3389/fmed.2021.741298

**Published:** 2021-09-08

**Authors:** Min Du, Liyuan Tao, Jue Liu

**Affiliations:** ^1^Department of Epidemiology and Biostatistics, School of Public Health, Peking University, Beijing, China; ^2^Research Center of Clinical Epidemiology, Peking University Third Hospital, Beijing, China; ^3^Institute for Global Health and Development, Peking University, Beijing, China; ^4^National Health Commission Key Laboratory of Reproductive Health, Peking University, Beijing, China

**Keywords:** vaccine hesitancy, COVID-19, Chinese, reproductive women, risk perception

## Abstract

**Background:** This study aimed to explore the association between risk perception and coronavirus disease 2019 (COVID-19) vaccine hesitancy among reproductive women in China to supplement limited studies in this area.

**Methods:** From December 14, 2020, to January 31, 2021, an anonymous cross-sectional online survey was conducted on COVID-19 vaccine hesitancy for children among reproductive women in China. We assessed risk perception, including perceived susceptibility, severity, barriers, and benefits using the health belief model, and then classified each variable into three groups (low, moderate, and high) based on tertiles. Information on sociodemographic characteristics, health status, and knowledge of COVID-19 was also collected. The Pearson χ^2^-test was used to compare vaccine hesitancy among the above mentioned factors. Logistic regression models were used to calculate the adjusted odds ratio (aOR) of risk perception related to vaccine hesitancy after controlling for the above covariates.

**Results:** Among 3,011 reproductive women, 8.44% (95%CI: 7.44. 9.43) had COVID-19 vaccine hesitancy. Vaccine hesitancy was observed more in women who lived in eastern China (11.63%), aged >45 years (12.00%), had a lower than high school education level (12.77%), and a low score on knowledge of COVID-19 (12.22%). Vaccine hesitancy was associated with lower perceived susceptibility (moderate: aOR = 1.72, 95%CI: 1.17–2.54, *P* = 0.0061; low: aOR = 2.44, 95%CI: 1.60–3.70, *P* < 0.0001), high perceived barriers (aOR = 2.86, 95%CI: 1.57–5.22, *P* < 0.0001), and lower perceived benefit (moderate: aOR = 3.29, 95%CI: 2.30–4.70, *P* < 0.0001; low: aOR = 4.59, 95%CI: 2.98–7.07, *P* < 0.0001), but not with perceived severity.

**Conclusions:** Although the proportion of COVID-19 vaccine hesitancy for children among Chinese reproductive women was <1 out of 10, to improve COVID-19 vaccine hesitancy, our findings suggest that tailored public health measures are needed to increase perceived susceptibility and benefit, and decrease perceived barriers among reproductive women.

## Introduction

As of July 2, 2021, coronavirus disease 2019 (COVID-19) is a major public health concern with more than one hundred million confirmed cases and three million deaths worldwide (1). Vaccination is considered the most economical and effective method for preventing infectious diseases. The World Health Organization (WHO) on June 30, 2021, reported that a total of 2,950,104,812 vaccine doses have been administered for COVID-19 (1). Although children with COVID-19 mainly have mild symptoms as compared to adults or are asymptomatic, some may be at risk for severe COVID-19, including a serious complication called multisystem inflammatory syndrome (2, 3). Therefore, COVID-19 vaccination is important for children. Currently, no COVID-19 vaccines are authorized for use among children aged <12 years, but the safety and efficacy of vaccines for children aged 6 months−17 years have been evaluated (4–6). The mRNA vaccine developed by Pfizer showed 100% efficacy and robust antibody responses among children aged 12–15 years (5). Han et al. reported that CoronaVac developed by Sinovac Life Sciences (Beijing, China) was well-tolerated and safe, and induced humoral response among healthy participants aged 3–17 years in China (7). Therefore, it is now essential to focus on the prospects of COVID-19 vaccination and the possible influencing factors in pediatric populations.

According to the WHO's Strategic Advisory Group of Experts, vaccine hesitancy is defined as the delay in acceptance or refusal to vaccinate oneself despite the availability of vaccination services (8). Vaccine hesitancy has been one of the ten threats to global health in 2019 (9). Children often rely on parental guidance and decision making, so reducing caregivers' vaccine hesitancy is a key point in achieving higher vaccination coverage among children in the future (10). Previous studies have investigated COVID-19 vaccine hesitancy in children. Skjefte et al. reported that only 69.2% of women indicated an intention to vaccinate their children across 16 countries (11). The percentage of parental COVID-19 vaccine hesitancy was 9.9% in Bologna, Italy (12), 10.9% in England (13), 20 and 27% in America (14, 15), 35% in British Columbia (16), 39.2 and 46.1% in Ankara (17, 18), and 49% in Germany (19). Investigations on COVID-19 vaccine hesitancy for children in lower middle income countries (LMICs) were limited. Skjefte et al. found that the proportion of COVID-19 vaccine hesitancy for children was below 15% in India and 30% in Philippine (11). Carcelen et al. reported that 8% of the caregivers had unwillingness of COVID-19 vaccination for their children in Zambia (20). Few researchers have investigated parental COVID-19 vaccine hesitancy in some provinces or territories of China. The proportion of parental unwillingness to vaccinate their children against COVID-19 was 14.7 and 12.5% in Shanghai, China (21, 22), 27.3% in Shenzhen, China (23), and 40.7% in Wuxi, China (24). Numerous factors are independently associated with parental vaccine decision-making, including risk perception (14, 15, 25), lifestyle, knowledge of vaccines (19), parental education (25, 26), vaccines' country of origin (27), history of vaccination against influenza (16), and parental psychological distress (23). Risk perception is a subjective construction process comprising multiple dimensions, including judgments on the severity and controllability of risks (28). People may develop risk perception for potential or actual consequences and the controllability of the COVID-19 pandemic based on cognitive appraisal theory (29, 30). Some studies have explored the association between risk perception and COVID-19 vaccine hesitancy in children in the USA (14, 15, 25), but related studies are scarce in China. The unknown situation of COVID-19 vaccine hesitancy for children and possible influencing factors especially for risk perception, were crucial and urgent for formulating policies to promote vaccination among children.

In summary, the proportion of COVID-19 vaccine hesitancy among children in China still remains unclear. Additionally, to prepare for COVID-19 vaccination among children, the association of risk perception and vaccine hesitancy to children should be explored so that we can provide a reference for proposing relevant measures. We used a sample of reproductive Chinese women to estimate the proportion of COVID-19 vaccine hesitancy for children and examine the association between risk perception and vaccine hesitancy after controlling for sociodemographic characteristics, health status, and knowledge of COVID-19.

## Materials and Methods

### Study Design, Participants, and Sampling

This anonymous cross-sectional survey was conducted from December 14, 2020, to January 31, 2021, in China using a stratified random sampling method via an online survey company established in 2006: Wen Juan Xing (Changsha Ranxing Information Technology Co., Ltd., Hunan, China). Wen (31), a specialized data science company with a database covers factual and well-characterized personal information (e.g., sex, region, and age) of over 2.6 million Chinese respondents. We can use the platform to conduct stratified random sampling, recruit target participants, and distribute questionnaires. Many researchers have used the recorded information in the database to obtain a representative sample and collect data from cross-sectional studies to investigate people's attitudes (32–34).

We recruited target participants for this study in China as the following inclusion criteria: (1) women aged 18–49 years; (2) Chinese speakers; and (3) voluntary agreement to participate in the present study. Considering that the proportion of COVID-19 vaccine hesitancy for children was 12.5% (22), with the alpha set as 0.05 and the confidence interval width as 0.1p (0.0125), the sample size was 2,690 when using PASS for calculation. Besides, regarding the rate of uncompleted questionnaire was 10%, so we planned to recruit at least 3,000 participants using an online survey platform (Wen Juan Xing) in three stages. First, we divided target participants into three tiers by region (eastern, central, and western regions), and selected two provinces randomly from each region. Second, the sample size for each province was allocated in proportion to the population of each province according to the China Statistical Yearbook 2020 (35). Third, Wen Juan Xing randomly selected and recruited target participants according to the sample size requirements in the sample database via the Wen Juan Xing online platform. We used Wen Juan Xing to set up logical jumps and other checked steps between questions to reduce missed and wrong answers. At the same time, before the questionnaire was released, internal staff pre-answered the questionnaire to estimate the reasonable time for answering the questionnaire (3–10 min). The study was approved by the Ethical Committee of Peking University Third Hospital (IRB00006761-M2020528) and conducted in accordance with the Declaration of Helsinki. Informed consent was obtained from all participants.

### Assessment of Risk Perception

We estimated risk perception to COVID-19 vaccination using the survey tool which was commonly used in previous studies for vaccination intention based on Health Belief Model (HBM) with good internal consistency reliability (17, 36, 37). HBM is an appropriate theoretical framework for understanding vaccination intent and illustrating the factors influencing people's decision-making about vaccination which is important to improve health promotion and reduce the barriers to vaccination (17, 36–38). The HBM includes five dimensions (perceived susceptibility, perceived severity, perceived barriers, and perceived benefits, and cues to action) comprising nine questions. In the present study, we used seven questions of HBM which evaluated risk perception, including perceived susceptibility, severity, barriers, and benefits. Two questions evaluated perceived susceptibility of infection to themselves and their children (if they had any), one question evaluated perceived severity of infection, three questions evaluated perceived barriers (vaccine safety, effectiveness, and the possibility of infection after vaccination), and one question evaluated perceived benefits of vaccination (protective effects). Participants answered each question on a three-point Likert scale (“*very concerned or agree*”, “*concerned or not sure*,” and “*not concerned or disagree*”), which were assigned the scores of 3, 2, and 1, respectively. We classified the participants into three groups based on the summed score for each HBM dimension by tertiles, with the top 33.3% of the participants being assigned to the “high” group, bottom 33.3% assigned to the “low” group, and middle ones assigned to the “moderate” group. Questions related to the Health Belief Model dimensions in the questionnaire as shown in Supplementary File 1. We did a pilot testing using a convenience sample of 20 participants and calculated Cronbach's alpha index for different dimensions of the health belief model. Cronbach's alpha index was 0.81 (perceived susceptibility), 0.88 (perceived severity), 0.76 (perceived barriers), and 0.87 (perceived benefits), respectively, showing an adequate internal consistency reliability.

### Measurement of Vaccine Hesitancy for Children

The primary outcome was the attitude toward COVID-19 vaccination for children. The question “If you have children under 18 years old, would you be willing to vaccinate them against COVID-19, when the vaccine becomes available?” was required to be answered by participants. People who answered “no” to this question were categorized into the hesitancy group.

### Covariates

In addition to HBM and attitudes toward COVID-19 vaccination, the following three aspects were investigated in the structured self-administered online questionnaire: (1) sociodemographic characteristics, (2) health status, and (3) knowledge of COVID-19.

Sociodemographic characteristics included age group, region, education, occupation, and monthly household income per capita (RMB). Health status included gravidity, parity, history of chronic disease, and history of influenza vaccination. Knowledge of COVID-19 comprised six aspects: source of infection, route of transmission, susceptible population, common symptoms, high-risk population for severe illness and death, and individual preventive measures for infection. For every correct response, the respondent received a score of one; otherwise, they received a score of zero. Then, we divided the total knowledge score into three groups (low, moderate, and high) by tertiles.

### Data Analysis

Mean (standard deviation; SD), frequencies and percentages were used to describe continuous and categorical variables, respectively. We compared the characteristics of participants with COVID-19 vaccine hesitancy using Pearson's χ^2^-test. The crude odds ratios (cORs) and adjusted odds ratios (aORs) of vaccine hesitancy in different risk perception groups were estimated using univariate and multivariate logistic regression models which were most frequently used statistical model for analyzing the relationship between outcomes and influencing factors (39, 40). We performed a sensitivity analysis by fitting different models to examine the robustness of the estimation. Model A was used as a univariate model. Sociodemographic characteristics—including age group, region, education, occupation, and monthly household income per capita, were adjusted in model B. Furthermore, all the covariates—including age group, region, education, occupation, monthly household income per capita, gravidity, parity, history of chronic disease, history of influenza vaccination, knowledge of COVID-19, and the other three risk perceptions—were adjusted in model C. Additionally, we supplemented model D which only adjusted the significant covariates and the other three risk perceptions based the Pearson's χ^2^-test.

We performed subgroup analyses on age group, region, education, occupation, monthly household income per capita, gravidity, parity, history of chronic disease, history of influenza vaccination, and knowledge of COVID-19 after adjusting for all the covariates. The heterogeneity test was used to examine differences between the groups. A *P* < 0.05, indicated statistical significance in this study. All analyses were conducted using SPSS 25.0, R 3.4.0, and Stata 16.0.

## Results

### Participants' Characteristics

In total, 3,150 participants out of 3,213 recruited participants completed the questionnaire (rate of completed questionnaire was 98.04%). We excluded 139 participants who completed the questionnaire in a short time (<1 min). Ultimately, our study included 3,011 eligible reproductive women. The average time for completing the survey was 8.93 min.

Of 3,011 women, 41.35% lived in central China, 61.04% were 30 years old or younger, and 94.92% had an education level of bachelor's degree or lower. The mean scores for perceived susceptibility, severity, barriers, and benefits were 4.14 (SD = 1.36), 2.59 (SD = 0.59), 5.22 (SD = 1.46), and 2.30 (SD = 0.66), respectively. Of the 3,011 reproductive women, 49.32, 64.70, 71.04, and 47.06% had moderate perceived susceptibility, high perceived severity, moderate perceived barriers, and moderate perceived benefit, respectively (Table 1).

**Table 1:** Risk perception among 3,011 reproductive women in China during COVID-19 pandemic.

**Risk perception**	***N***	**%**
**Perceived susceptibility**		
Low	798	26.50
Moderate	1,485	49.32
High	728	24.18
**Perceived severity**		
Low	164	5.45
Moderate	899	29.86
High	1,948	64.70
**Perceived barriers**		
Low	323	10.73
Moderate	2,139	71.04
High	549	18.23
**Perceived benefit**		
Low	349	11.59
Moderate	1,417	47.06
High	1,245	41.35

The total proportion of COVID-19 vaccine hesitancy for children was 8.44% (95%CI: 7.44, 9.43) among 3,011 reproductive women. According to χ^2^-tests, there were no differences in COVID-19 vaccine hesitancy according to income, gravidity, parity, history of chronic disease, history of influenza vaccination, and perceived severity among groups. Vaccine hesitancy was observed more in women who lived in eastern China (11.63%), aged >45 years (12.00%), had a below high school level education (12.77%), with a low score of knowledge on COVID-19 (12.22%). Additionally, vaccine hesitancy was more likely to be observed in women with low perceived susceptibility (11.03%), perceived benefit (14.94%), and perceived barriers (16.05%) (Table 2).

**Table 2:** COVID-19 vaccine hesitancy to children among 3,011 reproductive women in China by characteristics.

**Characteristics**	***N***	**COVID-19 vaccine hesitancy to children (%)**	**χ^2^**	***P***
**Total**	3,011	254 (8.44)		
**Sociodemographic characteristics**				
**Region**			18.582	<0.0001
Eastern	920	107 (11.63)		
Central	1,245	94 (7.55)		
Western	846	53 (6.26)		
**Age group (years)**			15.313	0.018
≤ 20	543	32 (5.89)		
21–25	712	48 (6.74)		
26–30	583	50 (8.58)		
31–35	469	48 (10.23)		
36–40	322	31 (9.63)		
41–45	207	24 (11.59)		
>45	175	21 (12.00)		
**Education**			10.88	0.012
Lower than high school	321	41 (12.77)		
High school or some college	886	77 (8.69)		
Bachelor's degree	1,651	121 (7.33)		
Postgraduate degree	153	15 (9.80)		
**Monthly household income per capita (RMB)**			3.496	0.321
≤ 3,000	1,562	122 (7.81)		
3,001–5,000	693	56 (8.08)		
5,001–10,000	571	58 (10.16)		
>10,000	185	18 (9.73)		
**Health status**				
**Gravidity**			5.419	0.067
0	1,607	118 (7.34)		
1	624	62 (9.94)		
≥2	780	74 (9.49)		
**Parity**			4.567	0.102
0	1,624	121 (7.45)		
1	825	81 (9.82)		
≥2	562	52 (9.25)		
**Chronic disease**			0.07	0.791
Yes	121	11 (9.09)		
No	2,890	243 (8.41)		
**History of influenza vaccination**			2.266	0.132
Yes	833	60 (7.20)		
No	2,178	194 (8.91)		
**Score of knowledge**			19.326	<0.0001
Low	769	94 (12.22)		
Moderate	1,337	93 (6.96)		
High	905	67 (7.40)		
**Risk perception**				
**Perceived susceptibility**			16.753	<0.0001
Low	798	88 (11.03)		
Moderate	1,485	128 (8.62)		
High	728	38 (5.22)		
**Perceived severity**			5.771	0.056
Low	164	20 (12.20)		
Moderate	899	85 (9.45)		
High	1,948	149 (7.65)		
**Perceived barriers**			39.372	<0.0001
Low	323	15 (4.64)		
Moderate	2,139	157 (7.34)		
High	549	82 (14.94)		
**Perceived benefit**			75.746	<0.0001
Low	349	56 (16.05)		
Moderate	1,417	154 (10.87)		
High	1,245	44 (3.53)		

### Association Between Risk Perception and COVID-19 Vaccine Hesitancy to Children

Models A, B, and C were established using logistic regression models, as shown in Table 3. In model A, without controlling for confounding factors, vaccine hesitancy for children was associated with lower perceived susceptibility (moderate: cOR = 1.71, 95%CI: 1.18–2.49, *P* = 0.0047; low: cOR = 2.25, 95%CI: 1.52–3.34, *P* < 0.0001; reference: high perceived susceptibility), low perceived severity (cOR = 1.68, 95%CI: 1.02–2.69, *P* = 0.0413; reference: high perceived severity), high perceived barriers (cOR = 3.61, 95%CI: 2.04–6.37, *P* < 0.0001; reference: low perceived barriers), and lower perceived benefit (moderate: cOR = 3.33, 95%CI: 2.36–4.70, *P* < 0.0001; low: cOR = 5.22, 95%CI: 3.44–7.90, *P* < 0.0001; reference: high perceived benefit). After controlling for sociodemographic characteristics in Model B, the above associations remained stable. After controlling for all covariates, vaccine hesitancy was associated with lower perceived susceptibility (moderate: aOR = 1.72, 95%CI: 1.17–2.54, *P* = 0.0061; low: aOR = 2.44, 95%CI: 1.60–3.70, *P* < 0.0001; reference: high perceived susceptibility), high perceived barriers (aOR = 2.86, 95%CI: 1.57–5.22, *P* < 0.0001; reference: low perceived barriers), and lower perceived benefit (moderate: aOR = 3.29, 95%CI: 2.30–4.70, *P* < 0.0001; low: aOR = 4.59, 95%CI: 2.98–7.07, *P* < 0.0001; reference: high perceived benefit), but not with perceived severity. Model D also showed the similar results (Table 3).

**Table 3:** The association between risk perception and the risk of COVID-19 vaccine hesitancy to children among 3,011 reproductive women in China.

	**Model A**		**Model B**		**Model C**		**Model D**	
	**Odds ratio** **(95% CI)**	***P*** **-value**	**Adjusted odds ratio** **(95% CI)**	***P*** **-value**	**Adjusted odds ratio** **(95% CI)**	***P*** **-value**	**Adjusted odds ratio** **(95% CI)**	***P*** **-value**
**Perceived susceptibility**								
Low	2.25 (1.52, 3.34)	<0.0001	2.32 (1.56, 3.46)	<0.0001	2.44 (1.60, 3.70)	<0.0001	2.37 (1.57, 3.58)	<0.0001
Moderate	1.71 (1.18, 2.49)	0.0047	1.75 (1.20, 2.54)	0.0037	1.72 (1.17, 2.54)	0.0061	1.69 (1.15, 2.49)	0.0075
High	Reference		Reference		Reference		Reference	
**Perceived severity**								
Low	1.68 (1.02, 2.76)	0.0413	1.73 (1.04, 2.86)	0.0346	1.38 (0.81, 2.37)	0.2351	1.47 (0.87, 2.50)	0.1530
Moderate	1.26 (0.95, 1.67)	0.1035	1.25 (0.94, 1.66)	0.1175	1.06 (0.78, 1.42)	0.7251	1.10 (0.82, 1.48)	0.5333
High	Reference		Reference		Reference		Reference	
**Perceived barriers**								
Low	Reference		Reference		Reference		Reference	
Moderate	1.63 (0.94, 2.80)	0.0792	1.59 (0.92, 2.74)	0.0974	1.09 (0.62, 1.93)	0.7550	1.09 (0.62, 1.91)	0.7709
High	3.61 (2.04, 6.37)	<0.0001	3.76 (2.12, 6.68)	<0.0001	2.86 (1.57, 5.22)	<0.0001	2.84 (1.56, 5.15)	0.0006
**Perceived benefit**								
Low	5.22 (3.44, 7.90)	<0.0001	5.19 (3.41, 7.90)	<0.0001	4.59 (2.98, 7.07)	<0.0001	4.57 (2.97, 7.03)	<0.0001
Moderate	3.33 (2.36, 4.70)	<0.0001	3.36 (2.37, 4.75)	<0.0001	3.29 (2.30, 4.70)	<0.0001	3.21 (2.25, 4.57)	<0.0001
High	Reference		Reference		Reference		Reference	

*Model A is a univariate model. Sociodemographic characteristics were adjusted in Model B. All covariates were adjusted in Model C, including region, age group, education, occupation, monthly household income per capita, gravidity, parity, history of chronic disease, history of influenza vaccination, knowledge of COVID-19, and the other three risk perceptions. Model D only adjusted the significant covariates including region, age group, education and knowledge of COVID-19, and the other three risk perceptions based the Pearson's χ^2^-test*.

**Figure 1:**
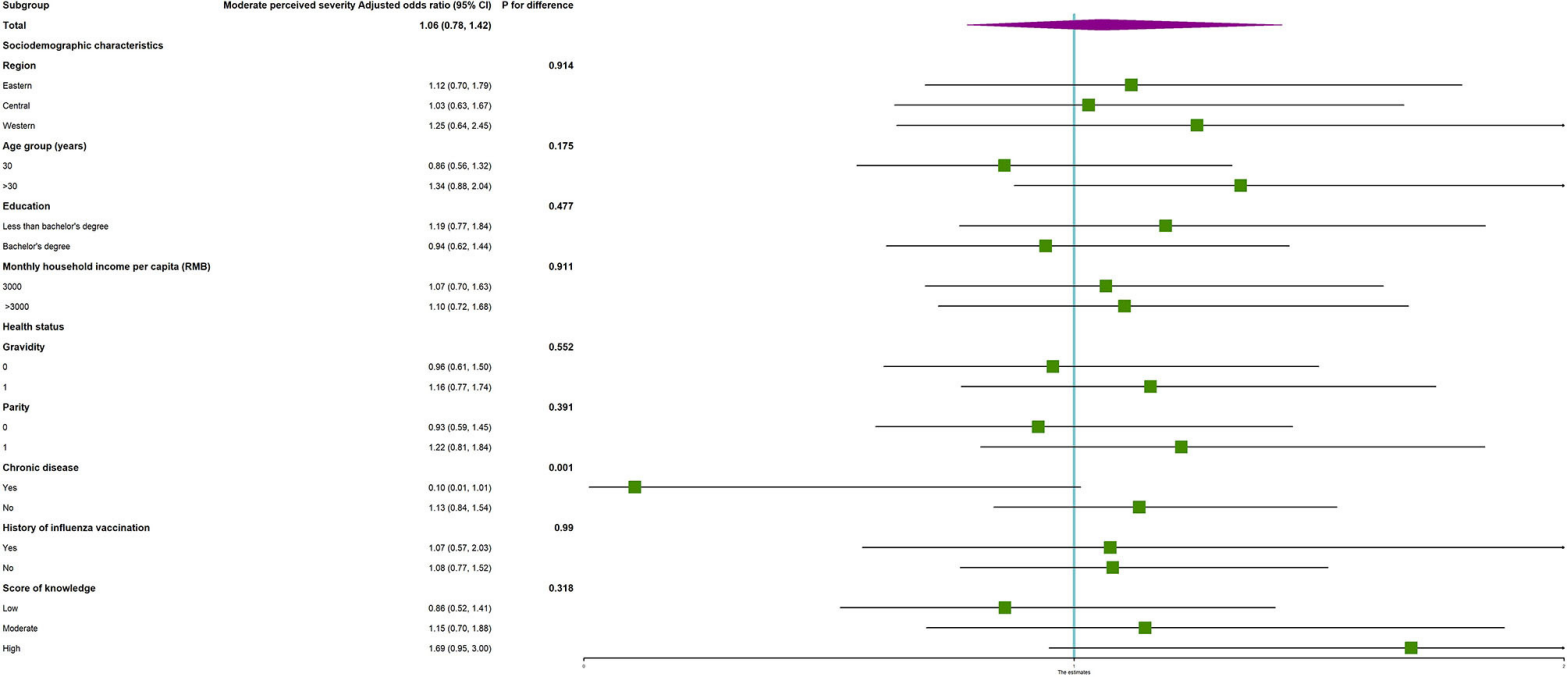
The subgroup analysis on the association between risk perception and the risk of COVID-19 vaccine hesitancy to children among reproductive women with moderate perceived severity.

Subgroup analysis showed no interactions in most subgroups (Supplementary Table 1). Regarding perceived severity, the vaccine hesitancy was more likely occurred among women with moderate perceived severity who had chronic diseases, as compared to those who had no chronic diseases (*P* for difference = 0.001, as shown in Figure 1), but vaccine hesitancy was not associated with moderate perceived severity in women with or without chronic diseases (Table 3).

## Discussion

As of July 2, 2021, COVID-19 is still a pandemic worldwide, with more than one hundred million confirmed cases and three million deaths worldwide (1). As of June 30, 2021, a total of 2,950,104,812 vaccine doses have been administered globally (1). The National Health Commission of the People's Republic of China reported that as of July 3, 2021, more than one billion vaccine doses have been administered to adults (41). Children are a susceptible population, however, no COVID-19 vaccines have been authorized for use among children aged <12 years (4–6). At the initial stage of COVID-19 vaccination in China, children were not a priority population for vaccination, with the completion of COVID-19 vaccination for adults, vaccination for children had been considered. CoronaVac has a well-tolerated, safe, and induced humoral response in Chinese children, according to a double-blind, randomized, controlled, phase 1/2 clinical trial (7). Therefore, on June 11, 2021, the Joint Prevention and Control Mechanism of the State Council announced that Chinese authorities have approved CoronaVac for emergency use in children aged 3–17 years, and experts are discussing formulating specific policies for vaccination (42). On July 20, 2021, Beijing Center for Disease Prevention and Control announced that COVID-19 vaccination for children aged 12–17 years old had been started in Beijing, China (43). However, the willingness to vaccinate children is not yet known. Moreover, the possible influencing factors, particularly, risk perception, were unclear. The unknown situation of the willingness to vaccinate children and possible influencing factors were crucial and urgent for formulating policies to promote vaccination among children. To our knowledge, this is the first online survey that investigated the proportion of COVID-19 vaccine hesitancy among children and examined its association with risk perception among reproductive women in six provinces of China.

We found that COVID-19 vaccine hesitancy to children associated with lower perceived susceptibility, high perceived barriers, and lower perceived benefit, but not with perceived severity. Some studies have reported that perceived higher risk of infection (34, 44), and lower perceived benefits and higher perceived barriers (36) among adults were associated with a high vaccine hesitancy. Our study found that vaccine hesitancy in children was associated with lower perceived susceptibility, perceived barriers, and perceived benefits in China, which is in line with other similar studies in the United States (14, 15, 25). Viswanath et al. found that perceived susceptibility (OR = 2.26, 95% CI: 1.56–3.27) to COVID-19 was associated with vaccine uptake for vaccinating those under one's care in the United States (25). Compared to the low perceived threat group, willingness to get children vaccinated was higher among those with high perceived threat (perceived severity and perceived susceptibility) (OR = 1.82, 95% CI: 1.21–2.72) (15). Thunstro¨m et al. reported that a higher degree of infectivity of the coronavirus may influence vaccine intentions (14). Additionally, our study found that perceived severity was not associated with vaccine hesitancy among children. Unlike Viswanath et al.'s findings (25), Thunström et al. (14) reported no statistically significant effect on parental vaccine intentions based on the severity of COVID-19. A systematic review regarded the link between perceived severity of illness as tenuous (45). Therefore, more research is needed to confirm the association between the perceived severity and COVID-19 vaccine hesitancy. Taken together, our findings suggest that elevating perceived susceptibility to COVID-19 and benefit of receiving the COVID-19 vaccine, while decreasing perceived barriers of receiving the COVID-19 vaccine, is an effective way to prevent COVID-19 vaccine hesitancy in children in China.

In our study, 26.50, 18.23, and 11.59% still had low perceived susceptibility, high perceived barriers, and low perceived benefits based on the summed score for each HBM dimension by tertiles. Taking measures and establishing programmes to increase perceived susceptibility and benefit and decrease perceived barriers is essential. Perceived barriers were evaluated by three questions including vaccine safety, effectiveness, and the possibility of infection after vaccination. Many studies also found that doubts regarding vaccine safety and efficacy were the main reasons for vaccine reluctance (11, 14, 18, 21, 22, 46, 47). Therefore, healthcare providers should use face-to-face education, autodialers, mail, and text messages to emphasize the COVID-19 pandemic situation, the benefits of vaccination, and the safety and efficacy of vaccines, to address parental concerns about vaccines. Moreover, training for healthcare providers, support from health authorities, and related media and social media channels should promote vaccinations (48, 49).

According to our investigation, the proportion of COVID-19 vaccine hesitancy among reproductive women in China was 8.44%, which was lower than that in some provinces or territories of China (21–24). For example, 87.5% would accept a vaccine with the most ideal attributes for their child, while with the least ideal attributes, these numbers dropped to 31.3% in Shanghai, China (22). Xu et al. reported that the proportion of COVID-19 vaccine hesitancy for their children was 27.3% in Shenzhen, China (23). Only 59.3% of parents reported willingness to avail COVID-19 vaccine for their children in Wuxi, China (24). The differences may be related to many factors, including the characteristics of the survey population, survey time, and region. Additionally, the results of this study showed that vaccine hesitancy was more likely to be observed in women who lived in eastern China, aged >45 years, had a high school or lower level of education, with a low score on knowledge of COVID-19. Montalti et al. and Khubchandani et al. also found that the highest vaccine hesitancy rates were detected in guardians with low educational levels (12, 50). Kelly et al. found that older individuals' willingness to vaccinate was higher (15). In addition, people with low knowledge scores on COVID-19 may be less aware of the susceptibility to disease and the importance of vaccination. These findings suggest that there should be targeted interventions for older women with a low education level and score of knowledge on COVID-19 in eastern China to decrease the proportion of vaccine hesitancy to children.

Our study has some limitations. First, reproductive women responding to the survey did not completely represent caregivers' attitudes in China as some of them had no children. Additionally, although we recruited participants using stratified random sampling from an online platform in six provinces of China, the database of the online platform may use an opt-in recruitment strategy, then there was a possibility of selection bias and an under coverage of sample frame which means that the results were not nationally representative and may not be generalizable to all women in China. Second, risk perception which was estimated using HBM may not be comparable to the reported situation of risk perception from other studies that used other models. Finally, we could not control for the effect of public health measures on vaccine hesitancy in China. What's more, the age of children for those who have children was not investigated in our study, so the specific situation of vaccine hesitancy for different aged children was unclear.

## Conclusion

The proportion of COVID-19 vaccine hesitancy among Chinese reproductive women was <1 out of 10. Vaccine hesitancy was more likely to be observed in women who lived in eastern China, aged >45 years, had high school or lower level of education, with a low score of knowledge on COVID-19. Importantly, vaccine hesitancy in children was associated with lower perceived susceptibility, perceived barriers, and perceived benefits.

To improve COVID-19 vaccine hesitancy to children, our findings suggest that tailored public health measures are needed to increase perceived susceptibility and benefit, and decrease perceived barriers among reproductive women. Additionally, there should be target interventions for older women with a low education level and score of knowledge on COVID-19 in eastern China to decrease the proportion of vaccine hesitancy to children. These findings have important implications for proposing strategies on COVID-19 vaccination for children in the future.

## Data Availability Statement

The raw data supporting the conclusions of this article will be made available by the authors, without undue reservation.

## Ethics Statement

The studies involving human participants were reviewed and approved by Ethical Committee of Peking University Third Hospital (IRB00006761-M2020528). The patients/participants provided their written informed consent to participate in this study.

## Author Contributions

JL conceptualized and designed the study. LT and JL did data acquisition and writing—reviewing and editing. MD did data curation, formal analysis, visualization, and writing—original draft. All authors contributed to the article and approved the submitted version.

## Funding

This study was funded by the National Natural Science Foundation of China, grant number 72122001, the National Science and Technology Key Projects on Prevention and Treatment of Major Infectious Disease of China, grant number 2020ZX10001002, and the National Key Research and Development Project of China, grant number 2020YFC0846300.

## Conflict of Interest

The authors declare that the research was conducted in the absence of any commercial or financial relationships that could be construed as a potential conflict of interest.

## Publisher's Note

All claims expressed in this article are solely those of the authors and do not necessarily represent those of their affiliated organizations, or those of the publisher, the editors and the reviewers. Any product that may be evaluated in this article, or claim that may be made by its manufacturer, is not guaranteed or endorsed by the publisher.

## Abbreviations

COVID-19: coronavirus disease 2019
HBM: Health Belief Model
SD: standard deviation
cOR: crude odds ratio
aOR: adjusted odds ratios.
