# Supplementary material for: The Association Between Risk Perception and COVID-19 Vaccine Hesitancy for Children Among Reproductive Women in China: An Online Survey
Source: Front Med (Lausanne). 2021 Sep 8;8:741298. doi: 10.3389/fmed.2021.741298 (PMC8455903; doi:10.3389/fmed.2021.741298)
Supplement: Supplementary file 1 [file Data_Sheet_1.docx]

**Supplemental file 1. Questions related to the Health Belief Model dimensions in the questionnaire**

**Perceived susceptibility:**

1. To which extent are you concerned about the following statements?

|  | Very concerned | concerned | Not concerned |
| --- | --- | --- | --- |
| I’m concerned about myself getting COVID-19. | □ | □ | □ |
| I’m concerned about my children (if you have) getting COVID-19. | □ | □ | □ |

**Perceived severity**

|  | Agree | Not sure | Disagree |
| --- | --- | --- | --- |
| If a people gets COVID-19, they are more likely to have severe illness. | ○ | ○ | ○ |

**Perceived barriers**

| COVID-19 vaccination can cause a person to get sick with COVID-19. | ○ | ○ | ○ |
| --- | --- | --- | --- |
| COVID-19 vaccination is not safe. | ○ | ○ | ○ |
| Vaccine is not an effective way to prevent people from getting COVID-19. | ○ | ○ | ○ |

**Perceived benefits**

| Getting COVID-19 vaccine is benefit for people. | ○ | ○ | ○ |
| --- | --- | --- | --- |

**Supplemental Table 1 The subgroup analysis on the association between risk perception and COVID-19 vaccine hesitancy to children for reproductive women.**

| **Subgroup** | **Low perceived susceptibility** | **P for difference** | **Moderate perceived susceptibility** | **P for difference** | **Low perceived severity** | **P for difference** | **Moderate perceived severity** | **P for difference** | **Moderate perceived barriers** | **P for difference** | **High perceived barriers** | **P for difference** | **Low perceived benefit** | | **P for difference** | **Moderate perceived benefit** | | **P for difference** |
| --- | --- | --- | --- | --- | --- | --- | --- | --- | --- | --- | --- | --- | --- | --- | --- | --- | --- | --- |
| **Total** | 2.44 (1.60, 3.70) | | 1.72 (1.17, 2.54) | | 1.38 (0.81, 2.37) | | 1.06 (0.78,1.42) |  | 1.09 (0.62, 1.93) | | 2.86 (1.57, 5.22) | | | 4.59 (2.98, 7.07) | | | 3.29 (2.30, 4.70) | |
| **Sociodemographic characteristics** | | | |  |  |  |  |  |  |  |  |  |  | |  |  | |  |
| Region |  | 0.347 |  | 0.313 |  | 0.959 |  | 0.914 |  | 0.986 |  | 0.703 |  | | 0.738 |  | | 0.274 |
| Eastern | 3.85 (1.87, 7.91) | | 2.40 (1.20, 4.78) | | 1.42 (0.60, 3.37) | | 1.12 (0.70, 1.79) | | 1.06 (0.44, 2.52) | | 2.00 (0.77, 5.17) | | | 3.82 (1.97, 7.41) | | | 2.14 (1.24, 3.70) | |
| Central | 2.08 (1.06, 4.06) | | 1.69 (0.92, 3.08) | | 1.47 (0.57, 3.78) | | 1.03 (0.63, 1.67) | | 1.21 (0.46, 3.15) | | 3.74 (1.36, 10.25) | | | 5.97 (2.87, 12.41) | | | 3.89 (2.12, 7.16) | |
| Western | 1.42 (0.61, 3.33) | | 0.99 (0.46, 2.17) | | 1.80 (0.64, 5.07) | | 1.25 (0.64, 2.45) | | 1.09 (0.31, 3.92) | | 4.13 (1.12, 15.18) | | | 3.98 (1.48, 10.70) | | | 4.83 (2.18, 10.69) | |
| **Age group (years)** | | 0.517 |  | 0.483 |  | 0.45 |  | 0.175 |  | 0.369 |  | 0.338 |  | | 0.779 |  | | 0.677 |
| ≤30 | 2.12 (1.22, 3.70) | | 1.51 (0.90, 2.53) | | 1.57 (0.83, 2.98) | | 0.86 (0.56, 1.32) | | 1.66 (0.65, 4.27) | | 4.50 (1.71, 11.86) | | | 4.28 (2.35, 7.82) | | | 3.43 (2.08, 5.65) | |
| >30 | 2.88 (1.54, 5.41) | | 2.05 (1.14, 3.69) | | 0.96 (0.34, 2.68) | | 1.34 (0.88, 2.04) | | 0.79 (0.38, 1.63) | | 1.89 (0.84, 4.22) | | | 4.91 (2.59, 9.29) | | | 2.92 (1.74, 4.90) | |
| **Education** | | 0.433 |  | 0.684 |  | 0.567 |  | 0.477 |  | 0.589 |  | 0.236 |  | | 0.598 |  | | 0.08 |
| Less than bachelor’s degree | 2.08 (1.14, 3.79) | | 1.57 (0.92, 2.71) | | 1.65 (0.79, 3.46) | | 1.19 (0.77, 1.84) | | 0.89 (0.40, 1.99) | | 1.69 (0.71, 4.04) | | | 5.60 (2.82, 11.13) | | | 5.29 (2.95, 9.49) | |
| Bachelor’s degree | 2.98 (1.65, 5.38) | | 1.87 (1.07, 3.29) | | 1.16 (0.51, 2.60) | | 0.94 (0.62, 1.44) | | 1.28 (0.56, 2.91) | | 4.50 (1.91, 10.58) | | | 4.28 (2.41, 7.61) | | | 2.22 (1.40, 3.53) | |
| **Monthly household income per capita (RMB)** | | 0.857 |  | 0.603 |  | 0.487 |  | 0.911 |  | 0.999 |  | 0.759 |  | | 0.331 |  | | 0.879 |
| ≤3,000 | 2.23 (1.22, 4.08) | | 1.88 (1.09, 3.25) | | 1.16 (0.56, 2.38) | | 1.07 (0.70, 1.63) | | 1.08 (0.48, 2.46) | | 3.16 (1.35, 7.40) | | | 3.52 (1.87, 6.64) | | | 3.28 (1.97, 5.48) | |
| >3000 | 2.42 (1.36, 4.32) | | 1.51 (0.87, 2.63) | | 1.82 (0.81, 4.08) | | 1.10 (0.72, 1.68) | | 1.08 (0.49, 2.37) | | 2.55 (1.10, 5.94) | | | 5.67 (3.11, 10.32) | | | 3.10 (1.88, 5.11) | |
| **Health status** | |  |  |  |  |  |  |  |  |  |  |  |  | |  |  | |  |
| **Gravidity** |  | 0.417 |  | 0.738 |  | 0.832 |  | 0.552 |  | 0.313 |  | 0.261 |  | | 0.237 |  | | 0.798 |
| 0 | 1.95 (1.07, 3.55) | | 1.55 (0.90, 2.68) | | 1.42 (0.72, 2.80) | | 0.96 (0.61, 1.50) | | 2.03 (0.71, 5.80) | | 6.15 (2.11, 17.89) | | | 3.48 (1.85, 6.55) | | | 3.11 (1.85, 5.22) | |
| ≥1 | 2.85 (1.58, 5.13) | | 1.78 (1.03, 3.11) | | 1.24 (0.49, 3.16) | | 1.16 (0.77, 1.74) | | 0.70 (0.35, 1.41) | | 1.56 (0.72, 3.38) | | | 6.38 (3.44, 11.83) | | | 3.43 (2.07, 5.70) | |
| **Parity** |  | 0.544 |  | 0.772 |  | 0.883 |  | 0.391 |  | 0.315 |  | 0.249 |  | | 0.269 |  | | 0.901 |
| 0 | 2.06 (1.14, 3.72) | | 1.56 (0.91, 2.69) | | 1.40 (0.71, 2.75) | | 0.93 (0.59, 1.45) | | 2.03 (0.71, 5.77) | | 6.37 (2.19, 18.49) | | | 3.56 (1.90, 6.66) | | | 3.21 (1.92, 5.37) | |
| ≥1 | 2.72 (1.51, 4.91) | | 1.76 (1.01, 3.07) | | 1.27 (0.50, 3.23) | | 1.22 (0.81, 1.84) | | 0.70 (0.35, 1.42) | | 1.51 (0.69, 3.29) | | | 6.25 (3.36, 11.64) | | | 3.36 (2.02, 5.59) | |
| **Chronic disease** | | 0.934 |  | 0.907 |  | 0.781 |  | 0.001 |  | - |  | - |  | | 0.962 |  | | 0.852 |
| Yes | 6.59 (0.22, 200.02) | | 5.74 (0.24, 135.72) | | 0.39 (0.01, 14.71) | | 0.10 (0.01, 1.01) | | - | | - | | | 5.40 (0.42, 69.19) | | | 2.32 (0.27, 19.76) | |
| No | 2.40 (1.57, 3.66) | | 1.70 (1.15, 2.52) | | 1.44 (0.84, 2.49) | | 1.13 (0.84, 1.54) | | 1.01 (0.57, 1.78) | | 2.85 (1.57, 5.19) | | | 4.57 (2.94, 7.12) | | | 3.26 (2.26, 4.69) | |
| **History of influenza vaccination** | | 0.661 |  | 0.666 |  | 0.667 |  | 0.99 |  | 0.528 |  | 0.725 |  | | 0.969 |  | | 0.603 |
| Yes | 3.04 (1.22, 7.58) | | 2.14 (0.90, 5.10) | | 1.91 (0.65, 5.58) | | 1.07 (0.57, 2.03) | | 2.56 (0.59, 11.21) | | 4.34 (0.93, 20.27) | | | 4.68 (1.97, 11.13) | | | 2.73 (1.33, 5.62) | |
| No | 2.29 (1.43, 3.66) | | 1.65 (1.07, 2.55) | | 1.34 (0.72, 2.49) | | 1.08 (0.77, 1.52) | | 0.84 (0.45, 1.57) | | 2.58 (1.34, 4.97) | | | 4.58 (2.77, 7.56) | | | 3.42 (2.26, 5.17) | |
| **Score of knowledge** | | 0.169 |  | 0.296 |  | 0.691 |  | 0.318 |  | 0.696 |  | 0.877 |  | | 0.584 |  | | 0.741 |
| Low | 1.74 (0.87, 3.48) | | 1.72 (0.92, 3.22) | | 1.52 (0.73, 3.18) | | 0.86 (0.52, 1.41) | | 1.30 (0.43, 3.92) | | 2.63 (0.84, 8.23) | | | 3.62 (1.77, 7.41) | | | 2.68 (1.48, 4.85) | |
| Moderate | 2.79 (1.39, 5.60) | | 1.63 (0.83, 3.20) | | 1.37 (0.53, 3.51) | | 1.15 (0.70, 1.88) | | 0.71 (0.29, 1.75) | | 3.50 (1.35, 9.03) | | | 6.60 (3.21, 13.58) | | | 3.78 (2.02, 7.07) | |
| High | 6.60 (3.21, 13.58) | | 3.78 (2.02, 7.07) | | 0.52 (0.07, 4.00) | | 1.69 (0.95, 3.00) | | 1.28 (0.48, 3.43) | | 2.23 (0.75, 6.68) | | | 3.59 (1.44, 8.94) | | | 3.45 (1.77, 6.69) | |

^1^effect size was aOR and 95%CI
